# The Enabling Role of ICT to Mitigate the Negative Effects of Emotional and Social Loneliness of the Elderly during COVID-19 Pandemic

**DOI:** 10.3390/ijerph18083923

**Published:** 2021-04-08

**Authors:** Carmen Llorente-Barroso, Olga Kolotouchkina, Luis Mañas-Viniegra

**Affiliations:** 1 Department of Applied Communication Studies, Complutense University of Madrid, 28040 Madrid, Spain; 2 Department of Audiovisual Communication and Advertising, University CEU San Pablo, 28040 Madrid, Spain

**Keywords:** elderly, unwanted loneliness, emotional well-being, social isolation, pandemic, COVID-19, ICT (Information and Communication Technology), digital communication, focus group

## Abstract

(1) Background: The COVID-19 pandemic has been especially hard on the elderly owing to their particular vulnerability to the virus. Their confinement to prevent the spread of the virus resulted in social isolation, often linked to the unwanted loneliness that hinders their emotional well-being. The enabling capacity of ICT (Information and Communication Technology) to overcome the negative effects of this isolation requires special attention. The purpose of this research is to understand the impact of the use of ICT on the emotional well-being of elderly people during their confinement. (2) Methods: A qualitative exploration method based on four focus groups with elderly people aged 60 years or older and three in-depth personal interviews with experts in education of the elderly were carried out. (3) Results: Research results evidence a negative emotional impact of the confinement (lack of physical contact with their loved ones, fear and uncertainty, feeling of loneliness, sadness at the loss of family members) on the emotional well-being of study participants. Furthermore, the operational capacity of ICT to prevent infection, as well as their positive emotional and humanizing role in providing access to entertainment and hobbies, and in improving self-esteem was also acknowledged. (4) Conclusions: ICT have become a valuable ally for elderly people aged 60 years and older to mitigate the negative effects of social isolation and loneliness imposed by the confinement.

## 1. Introduction

The COVID-19 pandemic has revealed the vulnerability of elderly people and the lack of social mechanisms to prevent their social isolation and unwanted loneliness as a result of the confinement. The perception of aging among the people over 65 years old is usually associated to loneliness [1], one of the most widespread and relevant issues among this age group along with social isolation [2]. The confinement has intensified this perception, becoming a public health concern [3] and drawing attention from media and governments [4].

The linkages between social isolation, loneliness and both physical and mental health have significantly increased the number of studies since 2000 [5], as a result of the concern within the societies with rapidly aging populations [3,4]. Social isolation and loneliness have particularly detrimental effects on the physical and mental health of elderly people [5,6,7], affecting from one third to half of this social group [3,8]. Both the perception of social isolation linked to the perception of loneliness and the feeling of lack of social and family support linked to rare interactions and limited social networks lead to affective disorders fostering or worsening symptoms of anxiety and depression in elderly people [9,10]. Some studies suggest that the subjective social isolation (loneliness) contributes significantly more to symptoms of depression and sleeping disorders in elderly people than the objective social isolation (limited social network) [11,12]. Moreover, it has been acknowledged that elderly people with a pessimistic self-assessment of their health, often depressed or facing emotional issues are at greater risk of mortality [13]. While elderly people that enjoy their life, in general experience better health [14].

During the pandemic, some governments recommended self-isolation of their elderly populations, which severely limited their social contacts with family and friends, forcing their isolation and loneliness. The negative effects of this situation on their health require specific measures to be adopted [15]. Prevention is usually acknowledged as one of the best tools to mitigate the adverse effects of anxiety, stress, and social isolation, triggered by the COVID-19 pandemic [16]. The social connection among elderly people can prevent affective disorders evolving into depressed mood or anxiety [9]. The social support has a positive impact on the well-being of the elderly [17]. Among the young old people (aged between 60 and 74) three kinds of social support acting as protection factors to prevent social isolation can be observed. In order of importance, they are friends, neighbors and family. Whereas for oldest old (aged over 74), only the support from friends acts as a protection factor [18]. An inadequate social support as well as living alone are factors with indirect and negative impact on the quality of life of elderly people due to the linkage of these variables with the increased depression rate of this social profile [17]. Even though findings of Tanner, Martinez and Harris indicate that “living alone was not a significant predictor of depression” [10] (p. 239).

The interaction between people enabled by some technologies fosters new forms of socialization [19]. Thus, establishing, promoting and optimizing social contacts [8,20], ICT (Information and Communication Technologies) become valuable tools for the mental health of the elderly, as enablers of social connection [21]. The spectrum of technological options to address social isolation and loneliness ranges from social media and video conferencing, to the Artificial Intelligence (AI) apps for the elderly [22]. In particular, ICT play a potential prevention role due to their capacity to mitigate social isolation of the elderly, even though not to the same extent for everyone [6], due to the disparities in access to the Internet, as well as the level of digital literacy [15,23,24]. Overall, the access to technology of the older age population group is limited [25]. Furthermore, some studies have determined that educational level is a predictor of technological capacity of the elderly, identifying not only an increased use but also an enhanced use [24,26]. In fact, more technologically advanced profiles identified by Vulpe and Crăciun as digitally immersed communicators, correspond to people aged 65–74 years old, of high educational level with over 20 years of studies [26].

In the hardest moments of social isolation during the pandemic, the role of technology in establishing a closer and more efficient communication has been enhanced [27]. Apps such as Facebook or WhatsApp enable elderly people to establish contact or to improve their relationship with family, friends, former colleagues and acquaintances and to find new contacts to share their interests or needs, notwithstanding temporary and geographical barriers, fostering their social inclusion [6,20,27,28,29,30].

The use of ICT by elderly people mitigates their loneliness and social isolation and facilitates digital interactions that [6]

1. Connect them to the outside world, improving their well-being [31,32];
2. Provide them with social support [8,19,20];
3. Foster their more active social participation, renewing their hobbies and empowering them with skills [24];
4. Enhance their self-esteem and their capacity to face new challenges [32,33,34];
5. Empower them through tools that help them to take control of their lives with greater security [34].

Käll et al. [35] highlight the utility of the Internet for the development of the internet-based cognitive behavior therapy (CBT), capable of alleviating subjective mood of loneliness. In addition, AI tools such as robots or voice assistants can accompany and provide social support for the elderly [22]. 

Despite that, often the ICT are not adapted to the needs and desires of the elderly due to the exclusion of this age group from the design process and/or the stigma attached to aging [31,36]. Age discrimination (ageism) is aggravated in many of the mechanisms used by digital platforms, affecting accessibility and usability of sites [36] and harming people with a low level of digital literacy, usually the elderly [37]. Furthermore, the digital capital “is negatively related to age” [38] (p. 804), as evidenced by the persistent age-based digital divide [38,39]. In Spain, only 39 per cent of people aged 65–74 use the Internet every day to perform easy tasks [40]. Nevertheless, in recent years, many old people have shown interest in the Internet, in particular, in its capacity to facilitate communication with the family [27,41]. The varied nature of interests in technology reveals intra-generational digital divides that emphasize the heterogeneity between different groups of elderly Internet users [26,42], in such a way that groups with lower digital literacy are at risk of social exclusion [43,44,45].

In this context, the purpose of this research is to understand the experience of elderly people aged over 60 in Spain during one of the hardest confinements in Europe, with the use of the Internet and ICT as tools to address social isolation, loneliness and distance from their love ones. Accordingly, three research questions (RQ) are addressed:

RQ1. What are the negative effects produced by the COVID-19 confinement on the well-being of elderly people aged over 60?

RQ2. What was the role of ICT in their everyday experience during such a difficult emotional moment?

RQ3. What was the role of ICT in stimulating mental and physical activity of the elderly? 

## 2. Materials and Methods

To address these research questions, a qualitative exploration method based on four focus groups aimed at understanding the reliance upon ICT of elderly people aged over 60 years to overcome isolation (individual or collective) and, in occasions, the perception of loneliness during an emotionally difficult episode or, even a traumatic event.

The focus group is a qualitative explanatory tool that explores perceptions and attitudes of a small group of people that interact and discuss a topic allowing their considerations [46,47,48,49,50,51,52,53]. A high number of interactions taking place in the focus group [54] foster a participative climate encouraging people to discuss and reflect upon a given topic in depth [55]. It is important to consider the context of the focus group setting as it is a determinant factor for the discursive production and its socio-cultural meaning [56]. The diversity of perspectives and affective processes emerging within the group context allows to explore the nature of coincidences and discrepancies between participants [52,57,58]. For these reasons, it is considered the most appropriate technique to address the research questions.

### 2.1. Sample, Data Registration and Procedure

Four focus groups were set up, considering the experiential affinity of their participants to facilitate the discussion of shared feelings [51,58]. The decision was made to set up small-size groups (6–8 participants) as they offer higher standards of emotional commitment and of interaction, in particular among the elderly [59]. In total, 27 people (n total) took part in focus groups, nine men and 18 women. This is a non-probabilistic convenience sample as participants were accessed through recommendation from colleagues and special institutions for the elderly. The selection criteria followed for the groups set up was the age of participants over 60 years old and their interest in autonomous, active and inclusive aging. Furthermore, each group was set up considering, on one hand, affinity criteria (level of digital literacy, place of residence, kind of isolation—individual or collective-), and on the other hand, some dissonance among participants to stimulate their sharing of different vital experiences or personal circumstances that could enrich the discussion and reflection of other participants (Table 1). The global approach to the sample design sought to ensure diversity of the previously defined variables in order to consider a wide range of situational circumstances and conditions as well as to ensure a potential richness of results. Considering these criteria, the following groups were set up (Table 1):

1. Group A: Formed by six people of high educational level, that were employed or are still employed by higher education institutions (universities or colleges). They are residents of urban areas in Spain and spent the confinement and some pandemic time living alone. This group was recruited with the support from the Complutense University of Madrid (UCM);
2. Group B: Formed by seven people aged over 60 years old, of high educational level and interested in lifelong learning. They are residents of urban areas in Madrid Autonomous Region, most of them from the capital of Spain. They spent the confinement and some pandemic time living alone. Almost all were employed in secondary education institutions or public administration and are retired now. This group was recruited with the support from the UCM—University for the Elderly;
3. Group C: Formed by six residents of Guadalajara city (Castile-La Mancha) of different educational background. More than 50% were employed (factory workers, secondary school teachers) and are retired now. This group was recruited with the support from the Center for Adult Education of Guadalajara CEPA Rio Sobre;
4. Group D: Formed by eight residents of a small village with population less than 200 people from Castile and León, of a medium-low and low educational background. More than 50% are employed and all of them have been employed in medium or low-skilled occupations (factory worker, welder, office assistant, caretaker, taxi-driver, personal assistant, shop assistant, cleaner). This group was recruited with the support from the Town Hall.

A semi-structured interview guide was developed before the focus groups took place with the purpose of orienting and prompting priority themes of the discourse accordingly to the queries raised, without hampering the discursive fluency and spontaneity of each group. The elaboration of this guideline addresses the need to homogenize questions, themes and procedures, while systematizing the development of all groups in order to identify consonance and dissonance among the participants [53]. Key thematic lines regarding the RQ, identified by the research group to guide the discourse, were as follows:

1. The negative impact of the worst moments during the COVID-19 pandemic on the well-being of the elderly aged over 60 years old;
2. The emotional, humanizing and operational role of ICT in enhancing well-being and mitigating fear of the elderly aged over 60 years old during the pandemic;
3. Learning ICT and ICT-enhanced learning as motivational triggers for the elderly during the pandemic.

The dynamic of the groups revealed a greater qualitative contribution of the discourse in groups A and B formed by people of higher educational level, while in groups C and D, the discourse was simpler and less profound, therefore requiring a greater motivation from the moderator. Overall, participants in each group agreed on almost all points because of their experiential affinities. Disagreements arose between groups or between participants in the same group when their personal circumstances had been different (in particular, Group D). Within the Group A, participation was fluid and the discourse rich in reflections. The participant GAS2 was the most dominant and the GAS6 was least participative. Within the Group B, GBS11 and GBS7 (the only male participant in this group) were the least involved female and male participants, while GBS10 was a slightly more dominant participant as compared to other active female participants. This group also revealed a rich discourse and a high involvement in suggested topics of conversation. Within the Group C, GCS16 was the least participative male member, while the most dominant was another male member GCS18. This group was the most uneven in the depth of contributions to the suggested topics. The Group D was the most difficult to manage for the moderator because of the less focused and more chaotic conversation on the suggested topics. GDS24 was the most dominant participant, while the participation of GDS26 and GDS27 was also noteworthy. The other extreme was the participant GDS20 who offered a very limited contribution, requiring appeals from the moderator. Table 2, Table 3, Table 4 and Table 5 in Section 3 (Results) outline a more explicit distribution of all the answers provided by different participants of the groups. 

In the light of the results of the focus groups, the importance of the latter thematic line for the people with lower level of digital literacy identified the need for semi-structured personal interviews with managers of educational institutions for the elderly. The purpose of those interviews was to know about their experience and motivational support for the elderly students during the pandemic. In particular, the perception of abilities of the elderly appears linked to their well-being in societies offering them skill-enhancing opportunities [60]. Furthermore, digital literacy of less skilled elderly people can contribute to the reduction of the intra-generational digital divide [61] through programs facilitating participative live to this group [62,63]. In this regard, educational institutions play an essential role. Thus, personal interviews were conducted with:

1. Bernardo Bienz [64], Executive President, Canal Senior (e-learning platform for the elderly);
2. María García-Carrillo [65], Director, Universitas Senioribus CEU (Private University for people aged over 55);
3. Beatriz Barrero [66], Coordinator, UCM-Universidad para los Mayores (Public University for people aged over 55).

The focus of these three personal interviews was on the third thematic line of the focus groups. A qualitative analysis was applied to assess key results.

### 2.2. Data Analysis

The length of each focus group was different depending on the discursive dynamics of their participants. The longest-lasting was of 02:23:06 while the shortest was of 01:21:08. All focus groups were conducted online, using the Blackboard Collaborate platform, under the safety and prevention protocol against COVID-19 by Spanish public authorities. All the discussions were recorded and transcribed for a further deep exploration of links between issues addressed [67]. Qualitative and inductive content analysis [68] was conducted using the ATLAS.ti 9 software (ATLAS.ti Scientific Software Development GmbH, Berlin, Germany) that allows the codification of the main themes linked to the guideline and of the sub-themes associated through inferential and objective criteria. Thus, a thematic analysis was conducted that allowed the categorization of data [69,70,71], identifying, codifying, and associating main thematic categories as well as new derivative themes [72]. The assessment of the themes in the four focus groups was conducted using the occurrences analysis of the global discourses that emerged in each group. The occurrence is the appearance of a specific theme or sub-theme in the discourse of the participant. The number of appearances of a specific theme in each group was registered with the cumulative number of occurrences by the ATLAS.ti 9 analysis. In Section 3 (Results), literal excerpts from different discourses are included with the purpose of clarifying or explaining specific aspects of each thematic category. 

The interviews were between 0:39:11 and 1:03:14 length and were also conducted online to follow the safety and prevention protocol. Given that those discourses were shorter and simpler, focusing on one thematic category, a qualitative analysis was conducted to identify mechanisms of emotional and motivational support implemented by two universities and the digital platform for their students and users. Literal excerpts were also extracted to clarify ideas.

## 3. Results

Table 2 outlines frequencies of themes and sub-themes in the discourses of the focused groups assessed with the ATLAS.ti software (ATLAS.ti Scientific Software Development GmbH, Berlin, Germany). In the following sections, a qualitative analysis is provided, specifying some important aspects of each of the thematic categories and their related ideas. 

### 3.1. The Negative Effect of the Hardest Moments during the COVID-19 Pandemic on the Well-Being of the Elderly Aged over 60 (RQ1)

These results are related to the most important thematic category in terms of its frequencies in different focus groups, with the exception of Group B (Table 2 and Table 3). To a great extent, this is due to a wide range of ideas linked to the emotional harm of the pandemic, in particular, during the strictest confinement. The distribution of the speeches on this theme and the related sub-themes is outlined in Table 3. 

#### 3.1.1. Lack of Physical Contact with Family and Friends, and Its Emotional Impact

Overall, all participants in the study acknowledged how difficult it had been for them not being able to have physical contact with their family, friends and relatives (34 total occurrences with a greater weight in Group D—14 reiterations (Table 2 and Table 3)): *“We are people used to […] engage with many people. These relationships have been greatly lost […] it´s been hard”* (GDS26). People with children and grandchildren highlight the emotional importance of having them nearby, being able to see them, embrace them, and share with them every moment of their lives. It was also particularly painful to sense the suffering experienced by their children unable to support them during the confinement: *“I know that my son suffered more from not being able to come to help me than I from not seeing him”* (GAS1). This concern is stronger among the participants with old parents and when the situation can be harmful for them both physically and emotionally: *“My […] mother is very old, she is 91, and I cannot see her and […] that hurts me a lot”* (GCS17).

#### 3.1.2. Perception of Loneliness during the Confinement

The perception of loneliness was not that dominant as might have been expected in different focus groups (15 total occurrences, Table 2 and Table 3). In fact, in Group A, notwithstanding the fact that all the participants spent their confinement alone, none of them felt lonely, to a great extent, on account of their high education level and digital literacy. Participants of other groups, with lower education level, felt different: *“You see, I´ve been alone for two months and a half, or three months […] not seeing anyone, alone”* (GDS27). When the feeling of loneliness was experienced, participants acknowledged that they felt better at the beginning of the confinement, expecting the situation would be temporary.

Among the most dramatic situations recalled was suffering caused by the loneliness of a relative dying in the hospital without the company of the loved ones: *“Sorry about getting emotional… It is hard and frustrating […] to assume that the person you love is there alone”* (GCS19).

Some participants, despite living with other people during the confinement, experienced a strange feeling of loneliness when they got sick and had to isolate in their room to prevent infecting others: *“To be there in the room, alone, the fever developing […] having them near, but not able to talk to them, that was quite hard”* (GDS25). The perception of loneliness was intensified with fear when they spent their illness entirely on their own. Faced with the loneliness imposed by the illness, believers relied on faith: *“I was feeling lonely, but […] I felt accompanied in my loneliness by someone who was there [by their faith]”* (GCS18).

#### 3.1.3. The Perception That Time Does Not Flow 

The participants in Groups B and D repeatedly (in 10 and 7 occasions, respectively) drew attention to the negative impact of time on the strength of their emotional well-being to cope with the confinement and the pandemic, when the social isolation was lifted (Table 2 and Table 3). In fact, many acknowledged that time management has got worse for them after the strictest confinement, due to the boredom: *“At the beginning we had the perception that it would be temporary temporal […] but […] it was getting longer”* (GBS13). Some participants acknowledge their feeling of increased sadness and anxiety, as they are still unable to meet their loved ones: *“We cannot see our children, we cannot embrace them […] This is the hardest part and the one that hangs heavy”* (GDS23). 

They try to cope with the negative perception about the passing of time through establishing routines and discipline, sometimes by means of physical and mental exercise (meditation): *“My daily meditation practice […] it was one of the pillars of my well-being”* (GBS9). Some specific activities such as sewing, crafts, reading, writing, solving sudokus, series, movies, Internet and ICT, played also a role of entertainment or distraction.

#### 3.1.4. Concern and Anxiety Motivated by Uncertainty and Fear

This theme has been the most frequent in group discussions (63 total occurrences, Table 2 and Table 3) with two main sub-themes: the anxiety raised by uncertainty (23 total occurrences, Table 2 and Table 3) and the anxiety raised by fear of getting sick (38 total occurrences, Table 2 and Table 3).

The shock caused by the situation led to concern and/or anguish that, in some cases, resulted in a mild form of anxiety. Many participants recognized that the need to understand what was happening, led them to a relentless search for information that, far from calming them down, increased their concern: *“Obliterating all other TV programs but the News reports […] instead of calming me down made me more worried […]”* (GAS2). The first-moment shock was followed by other concerns affecting their life, as they had known it, increasing their anguish: *“The first day I went out and saw all people wearing masks that was a shock*” (GBS9). That uncertainty resulted, in some cases, in poor concentration, blackouts, sleep disorders in participants of higher educational level: *“The curious thing was the lack of concentration, I suffered from that”* (GAS5). Overall, emotional control, acceptance and serenity helped to offset the adverse effect of uncertainty.

The fear of sickness, motivated by the vulnerability to the virus of many elderly people aged over 60, filled lives of all study participants, more intensely at the beginning of the pandemic because of the higher uncertainty: *“It’s scary, to go out and travel, I dare not imagine getting into a hotel”* (GCS15). That fear of infection significantly reduced their social relations even when they were allowed: *“Within my network, we didn’t reach the extremes, but there is fear […] we are not going out as before […] because of fear”* (GDS24). On some occasions, an excessive caution to avoid getting infected, led to nearly obsessive disinfection behavior that became more relaxed with the pandemic advancing: *“It was an obsession with cleaning and disinfection to avoid getting infected”* (GDS26). The fear of getting sick, of pain they could bear, or suspecting to be ill, without knowing for sure was hard to manage: *“I was very scared, every day I measured my temperature […] But I do not know if I got infected or not […] because nobody was under the follow-up”* (GDS21). Some of those who got infected, realized how close they had been to death, the feeling also experienced by those not infected: *“You feel that at any moment you can also die”* (GBS13). Almost all people that got sick had an intense fear of what was going to happen to their loved ones: *“The worst, […] I was mad about my mother, because you do not know if a person of 91 years old can pass it or not”* (GDS26). However, those who were not infected also developed a concern about friends and acquaintances suffering from sickness. To look at the situation from a positive perspective helped them to cope with fear: *“The positive effect of the fight for life became dominant and then I searched for practical solutions”* (GAS3). 

#### 3.1.5. The Profound Sense of Sadness Due to the Loss of Their Loved Ones

This sub-theme was particularly reiterative in groups of those who suffered the loss of their partners (Groups C and D, 13 and 8 occurrences, Table 2 and Table 3) and was also mentioned several times (7 occurrences) in the group with the largest social network (Group A). 

The usual sadness caused by the loss of a loved one reached a higher intensity during the confinement. The pain of loss was enhanced by the lack of a personal farewell and of shared grief with other relatives not living together: *“The worst, was to lose the granny [her loving mother] […] and not being able to give her the farewell”* (GDS23). Within this complex management of bereavements, feelings of guilt and helplessness also sometimes emerged, worsening the sadness: *“At the very beginning it was […] like a shade of guilt, same as it happened with HIV-AIDS issue”* (GAS2). On the other hand, a continuous passing away of acquaintances and relatives, although not too close, resulted in an ever-increasing pain in all participants. Not being able to join the loved ones of the passed away in sharing their grief: *“I didn’t feel a personal loss, but a similar shared loss […] I cried because it was very hard”* (GAS2).

During the strictest moments of the lockdown, the farewells were dramatic; burials happened without funerals and with a maximum of six direct family members (children). Not being able to attend the burial or celebrate the funeral was an emotional blow for those who suffered that experience: *“When Ángel [her husband] passed way, we were not able to embrace each other, the four of us alone… That was terrible”* (GCS19). In some cases, the burial was delayed for some months, leaving a deep emotional wound for a long time: *“Until we managed to bury three months later, it was that we had”* (GBS12). Once the lockdown was lifted, the funerals of their loved ones who passed away were hard to cope with emotionally, due to the severe restrictions imposed to prevent infections. Overall, emotions prevailed, addressing the mourning need of family and friends: *“This is what human beings are, all of a sudden […] four months without going out and disinfecting with bleach the doormat to enter the house with clean soles, and finally, […] to exchange affections we were running that risk […] and we gave farewell to our friend […] and we all felt much better than if we had not gone”* (GAS3). Nevertheless, those participants recognized the fear they suffered from experiencing that moment of emotional giving: *“The giving in that emotional moment was rewarded […] in the aftermath, let´s say, with some fear from being too reckless when we embraced each other”* (GAS4).

### 3.2. The Emotional, Humanizing and Operational Role of TIC during the Pandemic in Enhancing Well-Being and in Mitigating Fear of Elderly People Aged over 60 (RQ2)

This theme category was the most recurrent in Group B (31 occurrences) and the second most important in terms of its frequency in the discussions of all other groups (Table 2 and Table 4). This importance reveals the significant role of ICT in offsetting the negative effects of the confinement and of the pandemic on the well-being of the elderly. The distribution of the speeches on this theme and the related sub-themes is outlined in Table 4.

#### 3.2.1. Emotional Support, Fostering Contact with Their Loved Ones, Mediated by ICT

The communicative functionality of ICT has been comforting in bringing closer the majority of participants to their loved ones during the confinement (theme mentioned in 46 occasions, Table 2 and Table 4). For those who spent their confinement alone, technology has had a particularly important role in providing an escape from their loneliness: *“It´s been a strong support […] a bit of human touch to the imposed loneliness”* (GBS7). ICT has facilitated closeness with loved ones who were not able to meet each other because of the pandemic: *“My family is in Jaen […] and the contact is also through technology […] it made me feel closer”* (GAS6). Within the dynamic of emotional connections, video conferencing became a part of the participants’ lives, humanizing their hard emotional situation. Their grandchildren were the main driver for the screen-mediated contact: *“Another moment […] filling me most was […] connecting with my grandchildren over the video call. That was the driving force […] empowering me to move forward”* (GBS8). In some cases, technology increased contacts within the family as compared to the time before the pandemic: *“With my sister, we met over video call, and that was more frequent than we had met before”* (GAS2). 

The immediacy and the agility of WhatsApp made this app the most popular for daily connections between loved ones, in particular, the option of groups of friends was most important for people spending their confinement alone. The virtual connection also encouraged and comforted people who got infected or lost a loved one: *“When I started having fever […] in a group with my closest friends […] the affection you receive […] I believe WhatsApp was wonderful”* (GCS19). Zoom was another app widely used by some participants for video calls with family and friends. Social networking sites replaced at an emotional level, to some extent, the attendance to a funeral service and mitigated pain suffered by a woman who had lost her husband during the confinement: *“It was beautiful what we did […] on streaming […] a Mass that we were able to follow online all together, family and friends […] sharing comments; truly a very moving moment”* (GCS19). 

#### 3.2.2. Operational Role of ICT in Mitigating Fear through the Avoidance of Infection

The operational role of ICT enabled elderly people to develop activities online while reducing the risk of infection. This theme was particularly important for the participants with higher level of digital literacy (15 occurrences in Group A and 10 occurrences in Group B, Table 2 and Table 4). The majority of study participants recognized that they had developed this functional use of ICT before the confinement. However, all of them acknowledged its high utility during the lockdown. Almost all participants with higher educational level showed a higher digital literacy and used ICT more intensely. They tried to purchase groceries online; however, the collapse of the online delivery of supermarkets did not allow them to succeed in their endeavors: *“I tried to buy online at the beginning […] it was frustrating […] I remember the day I finally got access online at 10’00 am […] and at 8’00 pm I was finally able to make my purchase online”* (GAS3). None of the study participants with higher level of digital literacy experienced any issues with online banking during the confinement or with any other administrative procedure such as the annual tax declaration. The experience with online banking and online grocery purchase by the participants with lower level of digital literacy was more limited. Although, those who knew their limitations, acknowledged their utility during the pandemic: *“We learned Bizum […] we did not have it […] that´s the best of the world”* (GDS26).

Furthermore, three study participants who volunteer for Caritas highlighted the operational importance of WhatsApp for managing their volunteering with the vulnerable families suffering most extreme situation during the pandemic: *“Technology has been most important, the WhatsApp, in particular […] I was able to […] make […] them feel there was someone who cared about them”* (GCS14). 

#### 3.2.3. ICT as Entertainment and Distraction Tools during the Confinement

During the confinement, many study participants found out that ICT was a source of distraction and entertainment, helping them to pass their time while in isolation and enhancing their well-being. This role of ICT was particularly emphasized by those participants who spent their lockdown alone and were already retired (mainly in Group B, Table 2 and Table 4).

The devices have been their support to cope with their loneliness and isolation through distraction and entertainment: *“Technologies are very helpful and make you company, they distract you”* (GCS15). Some participants used them to play online with their loved ones, to watch series and movies, or to do some physical exercise as well-being routine: *“I followed a series of videos online on YouTube […] at a specific time […] to do some stretching”* (GBS8).

### 3.3. Learning ICT and ICT-Enhanced Learning as Motivational Triggers of Effort and Personal Autonomy of the Elderly (RQ3)

This theme category was the least recurrent in three of the four conducted focus groups (17 total occurrences, Table 2 and Table 5). The distribution of speeches on this theme and the related sub-themes is outlined in Table 5.

However, the importance of ICT as a motivational trigger for some participants during the lockdown motivated three interviews with experts in education for older people. The experts provided their views on the enabling capacity of ICT to enhance the well-being of this social group.

#### 3.3.1. Learning ICT and Self-Satisfaction 

Focus groups participants with lower level of digital literacy (Groups C and D, Table 2 and Table 5) admitted that the confinement made them learn something about ICT strengthening their self-esteem: *“[Something positive] we advanced a bit with technologies, we couldn’t have done half the things we’ve done so far”* (GDS26). The situation pushed them to strive and to renew their interest in ICT having seen how important they could be for them.

The interest of some study participants in learning ICT was also reflected in education initiatives for the elderly as highlighted by informants. Bienz [64] argues that the confinement led to a greater demand for online courses on technology topics. García Carrillo [65] stresses that technology has always been an essential element of the contemporary society; therefore, for the past eight years, her University for the elderly has been offering this kind of thematic courses. However, in the beginning, those courses were not highly demanded among their students due to *“some stress, fear and even embarrassment”* [65]. Offering free courses, they were able to encourage the participation of their students, reaching 7000 students who had their introduction to technology over seven years: *“That was not their idea, […] but […] when they were able to access […] free of charge, all their doubts vanished and they rushed in”* [65]. This experience is also shared by Barrero [66]; like the Complutense University of Madrid for the elderly, they also perceived that their courses focused on technology, albeit necessary, were not that appealing for their registered students. However, the need that emerged during the lockdown led them to create a Computer Club with streaming on YouTube, which is performing very well. 

#### 3.3.2. Use of ICT to Foster Hobbies and to Address Personal Concerns

ICT have been an essential pillar for some study participants to foster interests and hobbies through online learning in order to develop specific skills (in particular, those in Group B, Table 2 and Table 5): *“During the confinement, I followed several art courses […] I keep following them at least once a week”* (GBS10). Moreover, university education has played an important role for some participants: *“I am studying an undergraduate degree program at UNED (National University of Distance Learning), it was also useful […] I spent my time […] watching videos on the topic […] and once a week we had an online meeting with the lecturer”* (GDS22). Sometimes, hobbies were reinforced through social networking sites such as WhatsApp, Facebook or Instagram: *“Other groups [in WhatsApp] are focused on painting, and we set weekly challenges […] to paint something and to share the result with the group”* (GBS10). 

The Canal Senior (Senior Channel) launched the initiative “Active elderly people at home” that was joined by lecturers from university programs focused on the elderly: *“The response was truly good”* [64]. Furthermore, they tripled their educational offer and diversified contents in order to entertain their users [64]. In the case of Universitas Senioribus of the University CEU San Pablo and the Complutense University for the Elderly, the biggest challenge was the virtualization of their all-on-site courses in order to make company to their students during the hard confinement: *“It´s really been very difficult for elderly people, they were very sad […] our team had it very clear that we had to accompany our students”* [65]. In that way, those online connections with their universities became an exciting distraction tools: *“They told us it was a relief […] to have an oasis amid all that gloom, you see? a desert of loneliness”* [66].

## 4. Discussion

The major contribution of the results of this study lies in the identification of possibilities provided by ICT to the elderly that spent the pandemic in different emotional and social circumstances. In this regard, the research findings reveal the humanizing and emotional role of ICT, far beyond its operational capacities, in the context of the pandemic that affected well-being of elderly people. Furthermore, the operational capacity of ICT derived in learning ICT and ICT-enhanced learning, becoming a motivational trigger and improving mental well-being, as it was confirmed by experts interviewed for this research. 

The lack of physical contact with their loved ones as well as fears caused by uncertainty and the virus placed elderly people in the context of relative social isolation with a negative impact on their well-being. Nevertheless, social isolation and loneliness increase the risk of suffering from mental disorders such as depression [5,6,7,9,10], study participants did not develop any of those extreme conditions. This finding can be explained by the age of the participants in this study. The age over 75 years old is associated with a higher risk of developing depression [73]; however, only two study participants were aged over 75. Linnea et al. confirmed a lower prevalence of depression among younger elderly (60–66 years old), and its increase with age, notwithstanding the level of severity of depression [74]. In fact, Tazelaar et al. identified the 31.1% prevalence of the depressive symptoms among elderly people aged over 75, a significantly higher rate than among younger elderly [75]. 

Moreover, the support provided by ICT to the elderly might have been of great help, in particular, the emotional one, enabling them to feel united to their loved ones [30]. Furthermore, it facilitated a social connection, avoiding important affective disorders [9]. In regard to the emotional connection with their loved ones, WhatsApp has played the most prominent role for all the study participants. Although its usage was not restricted only to the family circle [20], as it was highlighted by previous research, but also widely used by single participants and their friends. 

The results also show some intra-generational inequalities in the use of ICT, identifying elderly people as heterogeneous group for their diverse online behavior [26,76]. Typically, elderly people with a higher educational level show a higher level of digital literacy [24,37] and have more resources to cope with a complex situation. Once again, education becomes a tool ensuring the enjoyment of life [14]. In fact, according to the results of this research, living alone was not the trigger of a lower well-being for the participants of higher educational background. These results are in line with findings of Martinez and Harris [10]. Meanwhile, participants with lower educational background and living alone showed a higher emotional weakness as happened with GDS27. These findings confirm the research results of Unalan et al. [17] regarding the link between low educational background and lower quality of life. The research findings show that the fact of living alone can become or not the trigger of loneliness, depending on the factors of educational background and digital literacy.

In general, ICT allow elderly people to cope with their loneliness and social isolation; however, not in all cases, they become suitable tools for all of them. Therefore, it is important to develop educational programs fostering digital literacy that suit each specific profile [6,23,24,32,62,63]. In fact, tools used by different digital platforms exclude elderly people, limiting their options and hindering digital equality [37]. In this regard, it is necessary to rethink the role of technology in age-friendly environments, to make it provide a committed support focused on social inclusion as a continuous and strategic process that enables an active and successful aging [20]. 

This study has confirmed again that the use of ICT empowers elderly people and strengthens their self-esteem, resulting in positive feelings and happiness [6,32]. However, these results should be considered within the exceptional circumstances imposed by the pandemic, highlighting the humanizing, emotional and social role of ICT. Elderly people with a higher level of digital literacy were able to take advantage through a more emotional and operational effective use of ICT. Those without that level of digital literacy acknowledged its importance and their interest in learning more, feeling very pleased with their progress during the confinement. However, the inter-generational support (mainly, from children) in the process of learning of those with lower level of digital literacy is still very important, to foster their independence and personal autonomy [27]. While the findings of this research show that this support has not been always available during the pandemic, fostering ICT-enhanced learning and compensating the technological stress with satisfaction of self-learning.

Notwithstanding the contribution of this research to the field of inclusive aging, supported by the social connection capacity of ICT, it is important to acknowledge its limitations. As an exploratory study focused on the national context, the research lacks the representative capacity of descriptive studies conducted in international context. Therefore, research findings should be further contrasted with transversal studies with representative samples, allowing the assessment of negative effects of social isolation on the emotional well-being of the elderly and the capacity of ICT to mitigate those effects. Furthermore, this research did not study the impact of socio-demographic variables such as sex or social status on the social isolation or the use of ICT to enhance the emotional well-being of the elderly. However, the variables sex, educational background and income have an impact on the quality of life of the elderly [17]. Furthermore, specific age groups were not considered by this research. Nonetheless, age is a predictor of lower digital literacy [37] that can explain some intra-generational divides in the effective use of ICT by elderly people, in order to avoid depressive moods in the framework of social isolation.

## 5. Conclusions

The findings allow a better understanding of the difficult emotional situation faced by elderly people aged over 60 who spent their confinement in different circumstances. In particular, concern and anguish caused by uncertainty and leading to anxiety and distress. The fear of sickness and the uncertainty made the majority of the study participants develop anxiety. Moreover, the negative emotional impact of the lack of physical contact with their loved ones is noteworthy, in particular, on elderly people with children and grandchildren living in other geographical regions. The perception of time has been different depending on the personal characteristics of each participant; managing their time was not that bad; however, this perception has worsened since the beginning of the pandemic. Loneliness was not a widely spread feeling among the study participants; however, when suffered, it was experienced in a different way, even though when they were accompanied and had to isolate to prevent infecting their partners (Group D). It is also worth highlighting the deep sadness of those who lost their loved partners and their difficulty in managing emotionally the bereavement without being able to give a farewell (Groups C and D).

The emotional and humanizing role of ICT was of extraordinary importance in the context of the most diverse circumstances of the study participants of the four focus groups. ICT enabled closeness to family and friends that were far away in the moment when it is important to feel close to the loved ones. However, within the group with the lower level of digital literacy (Group D) ICT was not able to mitigate the lack of physical and direct contact with children and grandchildren. Furthermore, the operational role of ICT in developing daily tasks online to avoid infection was revalued as those digital uses experienced growth. Although, this operational role of ICT was not that prominent, some participants who spent their confinement alone acknowledged the access to different formats of entertainment (series, games, etc.)

The importance of ICT to emotionally cope with and face the confinement and the pandemic led the study participants with lower level of digital literacy (Groups C and D) to self-learn some usages, resulting in increased self-esteem. Meanwhile, some elderly people with higher digital literacy, who spent the confinement alone, revalued the role of ICT to develop hobbies and address their personal interests, keeping them mentally active. In this regard, it is important to consider the contribution from the interviewed experts involved in educational programs for the elderly. They also perceived that interest in learning is essential nowadays. Moreover, online learning and social networking sites (Facebook, WhatsApp, Instagram) have fostered hobbies and interests of some elderly participants, facilitating their entertainment and strengthening their motivation to engage in enjoyable activities.

## Figures and Tables

**Table 1:** Focus groups setting (*n* = 27).

	Age	Sex	Confinement	Residence	Occupation	Studies	Digital Literacy	COVID	Death
Group A									
GAS1	77	F	Alone	A Coruña	Retired	Graduate	Medium	No	No
GAS2	72	M	Alone	Madrid	Retired	PhD	Very High	No	No
GAS3	69	F	Alone	Majadahonda	Active	Graduate	Very High	No	Friend
GAS4	67	M	Alone	Barcelona	Active	Graduate	Very High	No	Friend
GAS5	61	F	Alone	Oviedo	Active	PhD	Very High	No	Father-in-law
GAS6	60	M	Alone	Pobla de Vallbona	Active	PhD	Very High	No	No
Group B									
GBS7	77	M	Alone	Las Rozas	Retired	Graduate	High	No answer	No
GBS8	75	F	Alone	Madrid	Retired	Certified	High	No	No
GBS9	74	F	Alone	Madrid	Retired	Graduate	High	Yes	No
GBS10	72	F	Alone	Madrid	Retired	Graduate	High	No	No
GBS11	71	F	Alone	Madrid	Retired	Certified	Medium	No	No
GBS12	69	F	Alone	Madrid	Retired	Graduate	Very High	No	Cousin
GBS13	61	F	Alone	Madrid	Active	Graduate	High	No	No
Group C									
GCS14	73	F	Spouse	Guadalajara	Retired	Secondary	Medium	Yes	No
GCS15	70	F	Spouse	Guadalajara	Retired	Secondary	Medium–Low	Yes	Cousin
GCS16	69	M	Spouse	Guadalajara	Retired	Primary	Low	No	Cousin
GCS17	66	F	Spouse	Guadalajara	Retired	Graduate	High	No	No
GCS18	66	M	Congregation	Guadalajara	Active	Graduate	Medium–Low	Yes	Brother- in-law
GCS19	61	F	Spouse/Son	Guadalajara	Active	Graduate	Medium	Yes	Spouse
Group D									
GDS20	67	M	Spouse	Roda de Eresma	Retired	Primary	Low	No	No
GDS21	66	M	Spouse	Roda de Eresma	Retired	Primary	Low	Yes	Mother-in-law
GDS22	65	F	Son	Roda de Eresma	Retired	Secondary	Low	No	No
GDS23	63	F	Spouse	Roda de Eresma	Active	Secondary	Medium–Low	Yes	Mother
GDS24	62	F	Spouse	Roda de Eresma	Active	Secondary	Medium–high	No	No
GDS25	62	M	Spouse	Roda de Eresma	Active	Primary	Low	Yes	No
GDS26	61	F	Spouse	Roda de Eresma	Active	Secondary	Medium–Low	Yes	No
GDS27	60	F	Mother/Alone	Roda de Eresma	Active	Primary	Medium–Low	Yes	Mother

Source: Authors.

**Table 2:** Absolute occurrences or frequencies of the themes and sub-themes within the discourses of the four focus groups (*n* = 27).

	Group A	Group B	Group C	Group D	Total
1. Negative Effects of the Pandemic on the Well-Being of the Elderly Aged over 60 (T1)	35	26	43	43	147
1.1. Emotional impact of the lack of physical contact with their loved ones (S-T1.1)	8	4	8	14	34
1.2. Perception of loneliness during the confinement (S-T 1.2)	0	4	5	6	15
1.3. Negative perception about the passing of time (S-T1.3)	1	10	1	7	19
1.4. Concern and anxiety triggered by uncertainty (S-T1.4)	21	8	18	16	63
1.4.1. Anxiety about the uncertain situation (S-T1.4.1)	9	4	5	5	23
1.4.2. Anxiety about fear of sickness (S-T1.4.2)	11	5	11	11	38
1.5. Sadness at the loss of loved ones	7	3	13	8	31
1.5.1. Difficulty in managing the bereavement	3	2	5	2	12
2. Emotional, humanizing and operational role of ICT in enhancing well-being and mitigating fear of the elderly aged over 60 during the pandemic (T2)	28	31	22	18	99
2.1. Emotional support of ICT for the contact with their loved ones (S-T2.1)	11	15	11	9	46
2.2. Operational role of ICT in mitigating fear through the avoidance of infection (S-T2.2)	15	10	7	6	38
2.3. ICT as entertainment and distraction tools during the confinement (S-T2.3)	2	9	4	3	18
3. Learning ICT and ICT-enhanced learning as motivational triggers of effort and personal autonomy of the elderly (T3)	1	5	6	5	17
3.1. Learning ICT and self-satisfaction (S-T3.1)	1	1	5	4	11
3.2. Use of ICT to foster hobbies and to address personal concerns (S-T3.2)	0	5	0	1	6

Source: Authors, using ATLAS.ti.

**Table 3:** Distribution of occurrences per participant on the theme 1 and the related sub-themes (S-T1.1, S-T1.2, S-T1.3, S-T1.4, S-T1.4.1, S-T1.4.2, S-T1.5, S-T1.5.1) (Table 2).

	T1	S-T1.1	S-T1.2	S-T1.3	S-T1.4	S-T1.4.1	S-T1.4.2	S-T1.5	S-T1.5.1
Group A	35	8	0	1	21	9	11	7	3
GAS1	3	3	0	0	0	0	0	0	0
GAS2	11	1	0	0	8	3	4	3	0
GAS3	8	1	0	0	6	3	3	1	1
GAS4	6	1	0	1	3	2	1	1	1
GAS5	6	1	0	0	4	1	3	2	1
GAS6	1	1	0	0	0	0	0	0	0
Group B	26	4	4	10	8	4	5	3	2
GBS7	7	2	1	3	2	1	2	0	0
GBS8	3	0	1	1	1	1	0	1	0
GBS9	3	0	0	0	2	0	2	1	2
GBS10	4	0	0	2	1	0	1	1	0
GBS11	2	0	2	1	0	0	0	0	0
GBS12	2	1	0	1	0	0	0	0	0
GBS13	5	1	0	2	2	2	0	0	0
Group C	43	8	5	1	18	5	11	13	5
GCS14	9	2	1	0	6	2	3	0	0
GCS15	7	2	1	0	3	2	1	1	0
GCS16	4	1	0	1	1	0	1	1	0
GCS17	3	2	0	0	1	0	1	0	0
GCS18	14	1	2	0	6	1	4	5	2
GCS19	6	0	1	0	1	0	1	6	3
Group D	43	14	6	7	16	5	11	8	2
GDS20	2	0	0	2	1	0	1	0	0
GDS21	5	0	0	0	3	0	3	2	0
GDS22	3	2	0	1	0	0	0	0	0
GDS23	7	4	0	1	0	0	0	3	2
GDS24	9	1	1	2	7	3	4	1	0
GDS25	4	1	2	1	1	0	1	0	0
GDS26	6	3	0	0	4	2	2	0	0
GDS27	7	3	3	0	0	0	0	2	0

Source: Authors, using ATLAS.ti.

**Table 4:** Distribution of occurrences per participant on the theme 2 (T2) and the related sub-themes (S-T2.1, S-T2.2, S-T2.3) (Table 2).

	T2	T-S2.1	T-S2.2	T-S2.3
Group A	28	11	15	2
GAS1	2	0	1	1
GAS2	6	2	3	1
GAS3	3	1	2	0
GAS4	6	3	3	0
GAS5	7	3	4	0
GAS6	4	2	2	0
Group B	31	15	10	9
GBS7	4	2	1	1
GBS8	5	3	1	2
GBS9	6	4	2	3
GBS10	7	3	2	2
GBS11	0	0	0	0
GBS12	6	2	2	1
GBS13	3	1	2	0
Group C	22	11	7	4
GCS14	4	2	1	0
GCS15	4	1	1	3
GCS16	1	1	0	0
GCS17	5	3	1	1
GCS18	5	1	4	0
GCS19	3	3	0	0
Group D	18	9	6	3
GDS20	1	1	0	0
GDS21	0	0	0	0
GDS22	3	3	0	0
GDS23	0	0	0	0
GDS24	3	0	2	1
GDS25	1	1	0	0
GDS26	4	2	2	0
GDS27	6	2	2	2

Source: Authors, using ATLAS.ti.

**Table 5:** Distribution of occurrences per participant on the theme 3 (T3) and the related sub-themes (S-T3.1, S-T3.2) (Table 2).

	T3	T-S3.1	T-S3.2
Group A	1	1	0
GAS1	1	1	0
GAS2	0	0	0
GAS3	0	0	0
GAS4	0	0	0
GAS5	0	0	0
GAS6	0	0	0
Group B	5	1	5
GBS7	0	0	0
GBS8	3	0	3
GBS9	1	0	1
GBS10	1	0	1
GBS11	0	0	0
GBS12	0	0	0
GBS13	0	1	0
Group C	6	5	0
GCS14	1	1	0
GCS15	3	3	0
GCS16	0	0	0
GCS17	1	1	0
GCS18	1	0	0
GCS19	0	0	0
Group D	5	4	1
GDS20	0	0	0
GDS21	1	1	0
GDS22	1	0	1
GDS23	1	1	0
GDS24	0	0	0
GDS25	0	0	0
GDS26	1	1	0
GDS27	1	1	0

Source: Authors, using ATLAS.ti.

## Data Availability

The original contributions presented in the study are included in the article, further inquiries can be directed to the corresponding author.
